# Effect of Zeolite Incorporation on the Ion Release Properties of Silver-Reinforced Glass Ionomer Cement

**DOI:** 10.3390/biomimetics9060365

**Published:** 2024-06-17

**Authors:** Jessica Tan, Jessica Hao, David Vann, Krešimir Pavelić, Fusun Ozer

**Affiliations:** 1 School of Dental Medicine, University of Pennsylvania, Philadelphia, PA 19104, USA; jytan@upenn.edu (J.T.); haoje@upenn.edu (J.H.); 2 School of Arts and Sciences, University of Pennsylvania, Philadelphia, PA 19104, USA; 3 Faculty of Medicine, Juraj Dobrila University of Pula, 52100 Pula, Croatia; pavelic@unipu.hr

**Keywords:** glass ionomer cement, zeolite–clinoptilolite, dental materials, biomaterials

## Abstract

Background: Zeolite can release antimicrobial silver ions in a targeted and controlled manner for an extended time, selectively inhibiting the growth of pathogenic oral bacteria when added to dental materials. The objective of this study was to investigate the effect of the addition of zeolite to silver-reinforced glass ionomer cement on the release of silver ions over time. Methods: Five concentrations of silver–zeolite (0%, 0.5%, 1%, 2%, 4% wt) were incorporated into silver-reinforced GIC in the form of 10 mm × 2 mm circular disks (*n* = 5). The disks were incubated in deionized water at 37 °C and ion release from the samples was measured at 1, 2, 7, and 30 days after immersion by inductively coupled atomic emission spectroscopy. Results: Incorporating silver–zeolite increased silver ion release from silver-reinforced GIC disks compared to the control disks (*p* < 0.05), while incorporating zeolite alone had no effect. Higher concentrations of added silver–zeolite resulted in increased silver ion release. Sustained silver ion release was observed for up to 30 days. Conclusion: Adding silver–zeolite to silver-reinforced GIC may enhance its extended antibacterial effect in the oral cavity.

## 1. Introduction

Glass ionomer cements (GICs) are commonly used in restorative dentistry as lining, base, and filling materials [1,2,3]. They are formed from the reaction of water-soluble polyacrylic acid and basic ion-leachable aluminosilicate glass powder, mixed into a viscous paste that sets as a hard insoluble cement [4,5]. GIC chemically adheres to tooth structure via a chelation reaction between the carboxyl groups of polyacrylic acid and the calcium in the hydroxyapatite crystals of the tooth [1,2]. Due to its continuous fluoride ion release properties and biocompatibility, GIC has been demonstrated to reduce the incidence of recurrent caries [2,5]. Fluoride ions leach out of the glass particles of GIC following acid production by cariogenic bacteria in the oral cavity [6]. Fluoride ion release provides anticariogenic properties, including the inhibition of bacterial growth, reduction of bacterial acid production, and enhancement of enamel remineralization [5,6]. However, the limited amount of fluoride release from GIC has only a minimal antibacterial effect, which is not strong enough to inhibit bacterial growth or cause total bacterial destruction [7,8]. In addition, fluoride release from GIC only has a short-term effect, peaking at 24–48 h [9,10,11]. As a result, recurrent caries remains the main cause of failure for GIC restorations [12,13]. Combining GIC with a material with enhanced, long-term antibacterial activity could improve the effectiveness of GIC.

Zeolite is a porous aluminosilicate biomaterial with a similar composition to GIC [9,14]. It has a three-dimensional honeycomb-like structure with anionic pores that allows it to selectively exchange cations with the surrounding environment [9,15,16]. Previous studies have demonstrated the potential of zeolites to release antimicrobial cations in a targeted and sustained manner, making them promising additions to dental materials [16,17,18]. Zeolite exhibits a particularly strong affinity for silver ions, which allows it to electrostatically bind to them by up to approximately 40% by weight [19,20]. Consequently, zeolite can release these silver ions in a targeted and controlled manner for an extended time, selectively inhibiting the growth of pathogenic oral bacteria when added to dental materials [18,20]. 

Previous studies have demonstrated enhanced antimicrobial activity associated with the addition of silver nanoparticles to dental materials [21,22,23]. The proposed mechanism of this antibacterial effect is via the release of silver ions, which can invade bacterial cell walls and alter the permeability of bacterial cell membranes, resulting in cell death. Additionally, silver ions can generate reactive oxygen species, disrupt DNA replication, and inhibit the synthesis of essential bacterial proteins [24,25]. The incorporation of silver nanoparticles into dental materials has been demonstrated to inhibit the growth of cariogenic bacteria and reduce oral biofilm formation [26,27,28]. When released from these materials, silver ions exhibit bactericidal and anti-adhesive properties [29,30].

Silver-reinforced GIC material can release both fluoride and silver ions [31]. However, ion release studies have demonstrated that these materials can only release low levels of silver ions, with a compromised fluoride release [32,33,34]. The reinforcement of GIC with metal reduces the amount of surface area available for fluoride release, reducing the antimicrobial activity of the material [6]. Thus, enhanced silver ion release is desired to enhance the antimicrobial properties of these materials. We hypothesize that the incorporation of zeolite into silver-reinforced GIC will enhance the silver ion-release properties of silver-reinforced GIC for an extended period due to its ability to undergo selective cation exchange [15,16].

The antibacterial activity and compatibility of silver ions with zeolite make silver–zeolite a promising addition to GIC. Previous studies have shown that silver–zeolite is biocompatible and nontoxic, with significant potential for biomedical applications [8,16]. The use of a silver–zeolite coating on titanium alloy implant surfaces has demonstrated good biocompatibility [6]. Silver–zeolite-incorporated GIC has a similar biocompatibility to conventional GICs in cell cytotoxicity tests [17]. In addition, silver–zeolite has been demonstrated to enhance the antimicrobial properties of GIC in previous studies [9,14,17,22]. However, no studies have investigated the effect of silver–zeolite on silver-reinforced GIC. In this study, various concentrations of zeolite and silver–zeolite were incorporated into silver-reinforced GIC to determine their effects on silver ion release.

## 2. Materials and Methods

### 2.1. Preparation of Silver–Zeolite Powder

Silver-incorporated zeolite–clinoptilolite (silver–zeolite) was created using the ion exchange method [35,36,37]. Zeolite–clinoptilolite powder was added to 0.1 M AgNO_3_ solution, then shaken at room temperature in the dark for 24 h. The solution was then centrifuged for 15 min and the supernatant was collected. The precipitate was heated for 12 h. The remaining powder was collected.

To confirm that zeolite was able to take up silver ions using the ion exchange method, the silver ion concentration of the AgNO_3_ solution was measured via inductively coupled plasma atomic emission spectroscopy. The silver ion concentration decreased from 28,000 ppm to 20,300 ppm after zeolite was added, indicating that silver ions were absorbed by the added zeolite powder.

### 2.2. Preparation of GIC Discs

A previous study by our lab group tested the effect of adding zeolite to silver-reinforced GIC due to determine whether the porous nature of zeolite would compromise the physical properties of the material [38]. The addition of zeolite up to 4% by weight was found to have little detrimental effect on the physical properties of silver-reinforced GIC, allowing the material to retain its original surface hardness and flexural strength [38]. In addition, low concentrations of zeolite appear to have a negligible effect on the optical properties and light gray color of silver-reinforced GIC. Thus, concentrations of 0%, 0.5%, 1%, 2%, and 4% zeolite and 0%, 0.5%, 1%, 2%, and 4% silver–zeolite were selected for incorporation into Riva Silver, a silver-reinforced glass ionomer cement material.

One flat scoop of Riva Silver powder, as measured by the standard scoop provided in the Riva Silver Powder/Liquid Kit, was weighed on an analytical balance accurate to 0.0001 g. A portion of the measured powder, equivalent to the desired concentration of zeolite or silver–zeolite by weight, was removed. Zeolite or silver–zeolite was added to the powder until the original weight was achieved. The powders were mixed with a metal spatula until homogenous, then transferred to a mixing pad. One drop of Riva Silver liquid was added onto the mixing pad next to the powder, adhering to the powder–liquid ratio of approximately 0.300 g/0.043 (7:1) as provided by the manufacturer. The powder and liquid were mixed by hand with a metal spatula for 30 s until thoroughly combined. The sample mixture was immediately placed into a 3D-printed plastic mold to form circular disks 10 mm in diameter and 2 mm in thickness. To ensure uniformly smooth surfaces of the disk, the plastic mold containing the sample mixture was placed between two glass slabs and weighed down by a 300 g weight. The sample was left undisturbed for 6 min to allow for complete setting, then removed from the mold. A scalpel was used to trim any excess material from the periphery of the disks. Identical steps were followed for the remaining samples so that a total of five samples per concentration group per time point were created.

### 2.3. Ion Release Measurement

For a comparison of the difference between silver ion release from zeolite-incorporated GIC and silver–zeolite-incorporated GIC, prepared samples from each concentration group (*n* = 5) were immersed in 5 mL of deionized water and incubated at 37 °C for 48 h. This timeframe was chosen based on previous studies on conventional GICs that demonstrated their ability to release high amounts of fluoride up for to 48 h, followed by a gradual decrease in their release to a constant level thereafter [39,40]. Deionized water was selected as the incubation medium in order to isolate the effect of zeolite on silver ions specifically, since zeolite has been demonstrated to exchange other cations [41]. After 48 h of incubation, silver ion release was measured via inductively coupled plasma atomic emission spectroscopy (ICP-AES). Calibration curves were constructed using standard solutions to quantify the silver ion release from each sample.

For the measurement of extended ion release from silver–zeolite-incorporated GIC over time, prepared samples of silver–zeolite-incorporated GIC from each concentration group (*n* = 5) were immersed in 5 mL of deionized water and incubated at 37 °C for 1, 2, 7, or 30 days. After the designated incubation period, silver ion release was measured via ICP-AES using standardized calibration curves to quantify the silver ion release from each sample.

### 2.4. Statistical Analysis

Data sets were analyzed via one-way ANOVA tests at α = 0.05 to test for differences in silver ion release from the sample groups at each time point.

## 3. Results

### 3.1. Ion Release from Zeolite-Incorporated GIC vs. Silver–Zeolite-Incorporated GIC

**Figure 1:**
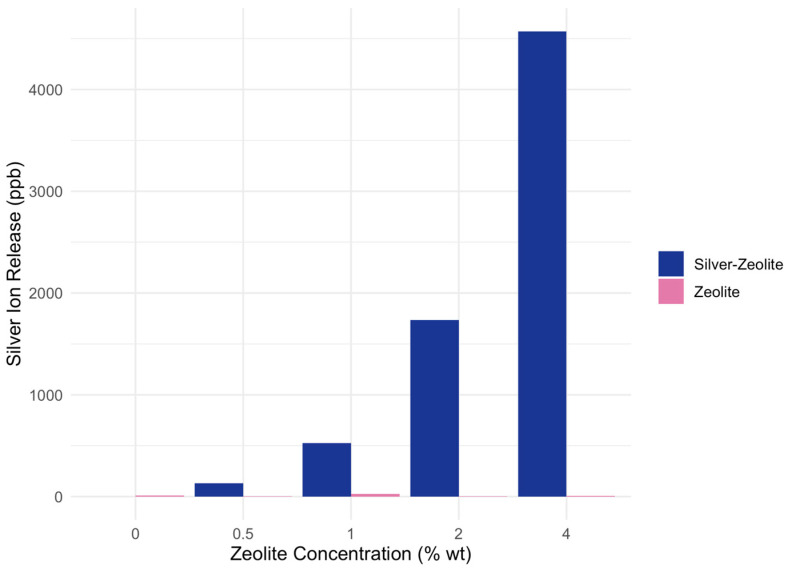
Mean silver ion release in parts per billion (ppb) from increasing concentrations of zeolite and silver–zeolite after 48 h of incubation.

Figure 1 shows the mean silver ion release for each concentration of zeolite and silver–zeolite, comparing data from 24 h after. The mean silver ion release from all concentrations of zeolite incorporated GIC discs (0.5%, 1%, 2%, and 4% wt) was compared to the control (0% wt) for zeolite-incorporated GIC discs and silver–zeolite-incorporated GIC discs (Table 1). The amount of silver ion release from all concentrations containing silver–zeolite (0.5%, 1%, 2%, 4% wt) showed significantly higher concentrations compared to the control (0% wt). The amount of silver ion release from all concentrations containing zeolite alone (0.5%, 1%, 2%, 4% wt) did not show significant difference from the control (0% wt).

### 3.2. Ion Release from Silver–Zeolite-Incorporated GIC over Time

**Figure 2:**
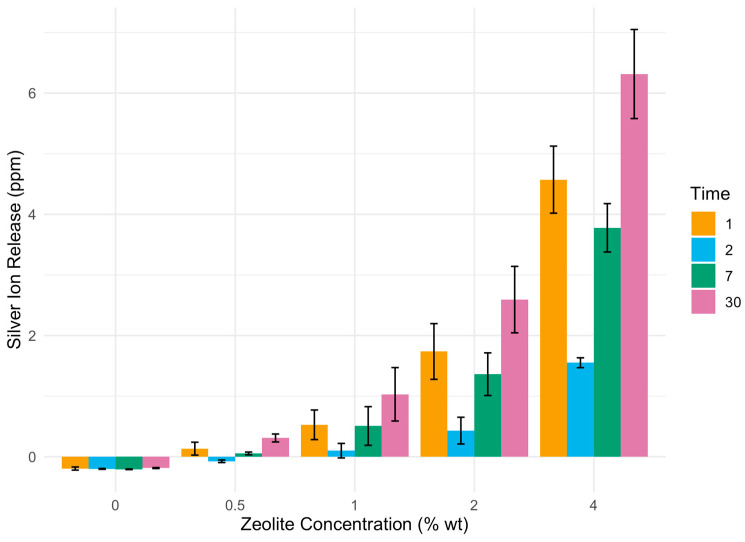
Silver ion release in parts per million (ppm) from increasing concentrations of zeolite over time, measured by inductively coupled atomic emission spectroscopy.

The ion release of silver ions from Ag-reinforced GIC was found to be significantly higher for all concentrations of added zeolite (0.5%, 1%, 2%, and 4% wt) compared to the control group without zeolite incorporation (*p* < 0.05). Furthermore, a concentration-dependent increase in silver ion release was observed, with higher concentrations of zeolite leading to enhanced ion release (Figure 2).

Silver ion release was evident at all measured time points. Silver ion release continued to increase for up to 30 days after immersion in each concentration group (Table 2 and Figure 2).

## 4. Discussion

This study investigated the effect of zeolite incorporation on the silver ion release properties of silver-reinforced GIC. The initial hypothesis posited that zeolite would enhance silver ion release due to its ion uptake and release capabilities. Contrary to our initial hypothesis, the results suggest that incorporation of zeolite alone did not enhance silver ion release from silver-reinforced GIC. This observation raises questions about the ability of zeolite to effectively uptake and release silver ions that are incorporated within silver-reinforced GIC. It is plausible that there may not have been enough silver ions in the silver-reinforced GIC for zeolite to take up. A previous study by Williams et al. demonstrated that only a small number of silver ions were released into deionized water when measured for up to one year [34]. Alternatively, zeolite may not be able to efficiently interact with silver ions within the GIC powder, limiting its potential to enhance silver ion release. However, Akgül et al. demonstrated the ability of zeolite to uptake and remove silver ions from aqueous solution [42]. At this time, no studies have explored the ability of zeolite to uptake silver ions from silver-reinforced GIC.

When zeolite was charged with silver ions to form silver–zeolite, its incorporation into silver-incorporated GIC did lead to a significant enhancement of silver ion release. This finding indicates that while zeolite alone may not be effective in promoting silver ion release from silver-reinforced GIC, the combination of silver-incorporated zeolite with GIC can enhance its ion release properties. This corroborates previous research demonstrating the potential of silver–zeolite-incorporated GIC to release silver ions in a controlled manner for up to 48 h [35,36]. This effect could be attributed to the extended release of silver ions that were absorbed into the anionic pores of zeolite during the ion exchange process [37].

The amount of silver ion release from silver-reinforced GIC was significantly higher for all concentrations of added silver–zeolite compared to the control. All tested concentrations (0.5–4% wt) were shown to maintain the physical properties of GIC in a previous study conducted in our laboratory [38]. In this study, higher concentrations of incorporated zeolite resulted in increased silver ion release. A similar study by Çinar et al. also demonstrates the increasing antibacterial effect of silver–zeolite-incorporated GIC with increasing concentrations of silver–zeolite (0.2–2%) [36]. Furthermore, a study by Hotta et al. showed that increasing the concentration of Ag-Zn–zeolite increased silver and zinc release from resin cement [43]. However, this increase did not correspond to increased antibacterial activity [43]. Thus, further research is needed on the antimicrobial properties of silver–zeolite-incorporated silver-reinforced GIC to confirm the clinical significance of the increased silver release noted in this study.

Increased silver release was observed at 30 days after immersion in each concentration group, indicating the potential for the extended-release properties of silver–zeolite-incorporated silver-reinforced GIC beyond the short-term 24–48 h fluoride release of conventional GICs [8,9,10]. The sustained release of silver ions from zeolite-incorporated GIC suggests its potential for longer-term antimicrobial activity. This extended antimicrobial property was also demonstrated in studies on silver–zeolite’s incorporation into other dental materials, including root end filling materials and acrylic resins [43,44,45]. Further research on the release of silver ions beyond 30 days is needed to determine the long-term antimicrobial ion release and mechanical properties of the zeolite-incorporated GIC.

Further research is needed to investigate the ion release properties of silver–zeolite-incorporated silver-reinforced GIC under varying experimental conditions. The release of ions from dental materials can be influenced by various factors, including pH, temperature, and the presence of other ions in the oral environment. A limitation of this present in vitro study is that the experimental conditions tested in this in vitro study do not match the true oral environment. In this study, the samples were incubated in deionized water, which is not representative of the diverse composition of ions present in human saliva. Zeolite can exchange monovalent and multivalent cations found naturally in saliva (e.g., Na^+^, H^+^, Ca^2+^, Cu^2+^), which may alter its silver ion uptake and release properties in the oral cavity [41].

In addition, further research is needed to investigate the effect of silver–zeolite-incorporated silver-reinforced GIC on dental biofilms. While several studies have demonstrated the antibacterial effect of GIC on planktonic bacteria on agar plates, there are limited studies on the effects on bacteria in biofilms [9,37]. Ge et al. demonstrates an enhanced killing of *S. mutans* and decreased bacterial metabolic activity in biofilms on silver–zeolite-incorporated GIC compared to the control [16]. El-Wassefy et al. showed a decrease in *S. aureus* biofilm formation with silver nanoparticle-integrated GIC [22]. No studies have investigated the long-term effect of adding silver–zeolite to silver-incorporated GIC on biofilm formation.

## 5. Conclusions

The incorporation of zeolite into silver-reinforced GIC significantly enhances the silver ion release properties, with higher concentrations of zeolite resulting in increased ion release. These findings clearly highlight the potential of zeolite as a controlled-release agent that can enhance the antimicrobial efficacy of dental materials. Therefore, our future studies will focus on evaluating the antimicrobial effectiveness of zeolite-incorporated GIC against a variety of oral microorganisms and its impact on biofilm formation, further elucidating its potential applications in preventive dentistry.

## Figures and Tables

**Table 1:** Mean silver ion release in parts per billion (ppb) from increasing concentrations of zeolite and silver–zeolite after 48 h of incubation.

	C (0%)	0.5%	1%	2%	4%
Zeolite	10	4	25	5	6
Silver–zeolite	0	113 *	527 *	1737 *	4572 *

* *p* < 0.05.

**Table 2:** Silver ion release in parts per million (ppm) from increasing concentrations of zeolite over time.

	C (0%)	0.5%	1%	2%	4%
24 h	−0.1945	0.1335 *	0.5274 *	1.7366 *	4.5722 *
48 h	−0.2000	−0.0745 *	0.1000 *	0.4212 *	1.5512 *
7 days	−0.2069	−0.0547 *	0.5075 *	1.3620 *	3.7776 *
30 days	−0.1867	0.3097 *	1.0305 *	2.5932 *	6.3150 *

* *p* < 0.05.

## Data Availability

Data are contained within the article.
